# Survival Benefit after Shifting from Upfront Surgery to Neoadjuvant Treatment in Borderline Resectable Pancreatic Cancer

**DOI:** 10.3390/biomedicines11082302

**Published:** 2023-08-18

**Authors:** Hyun Jeong Jeon, Soo Yeun Lim, HyeJeong Jeong, So Jeong Yoon, Hongbeom Kim, Sang Hyun Shin, Jin Seok Heo, In Woong Han

**Affiliations:** 1 Division of Hepatobiliary-Pancreatic Surgery, Department of Surgery, Kyungpook National University Chilgok Hospital, School of Medicine, Kyungpook National University, Daegu 41404, Republic of Korea; 2 Division of Hepatobiliary-Pancreatic Surgery, Department of Surgery, Samsung Medical Center, Sungkyunkwan University School of Medicine, Seoul 06351, Republic of Korea

**Keywords:** borderline resectable pancreatic cancer, neoadjuvant treatment, upfront surgery

## Abstract

According to the 2016 National Comprehensive Cancer Network (NCCN) guidelines, patients with borderline resectable pancreatic cancer (BRPC) should receive chemotherapy as the first-line treatment. This study examined the real-world survival benefits of modifying BRPC treatment guidelines. Patients treated for BRPC at a single institution from 2013 to 2015 (pre-guideline group) and 2017 to 2019 (post-guideline group) were retrospectively reviewed. According to the treatment method used, patients were classified into upfront surgery (US), surgery after neoadjuvant treatment (NAT), and chemotherapy only (CO) groups. Overall survival (OS) was compared according to period and treatment type. Factors associated with OS were analyzed using a Cox regression model. Among the 165 patients, 63 were in the pre-guideline group and 102 patients were in the post-guideline group. The median OS was significantly improved in the post-guideline group compared to the pre-guideline group (29 vs. 13 months, *p* < 0.001). According to the treatment method, the median OS of the NAT group was significantly longer than that of the US and CO groups (40 vs. 16 vs. 15 months, respectively, *p* < 0.001). In multivariate analysis, tumor size, differentiation, NAT, and perineural invasion were significant prognostic factors. NAT is an important treatment option for BRPC and increased patient survival in the real world.

## 1. Introduction

Pancreatic cancer is an aggressive malignant tumor, and its incidence has increased by 1% annually since the 2000s. However, the 5-year survival rate has increased by only 6–11% over the past few decades, showing a stagnant outcome compared to other cancers, including gastric cancer and colorectal cancer [1]. Surgical resection provides an opportunity for curative potential, but resectable cases account for only 20–25% at the time of diagnosis [2]. Chemotherapy is the mainstay treatment for metastatic and locally advanced pancreatic cancer (LAPC). The 5-year survival rate for advanced pancreatic cancer is 1–14%, which is associated with an abysmal prognosis.

Various efforts have been made to change the treatment paradigm and increase the survival rate of patients with pancreatic cancer. The introduction of combination therapy with 5-fluorouracil, leucovorin, oxaliplatin, and irinotecan (FOLFIRINOX) has shown improvements in chemotherapy efficacy [3,4]. In addition, several studies have reported cases of conversion surgery from LAPC after primary chemotherapy [5,6]. As the efficacy of chemotherapy has been proven, a multidisciplinary treatment approach that combines chemotherapy and surgery has become the mainstay for pancreatic cancer.

As part of this trend, the 2016 National Comprehensive Cancer Network (NCCN) guideline recommended surgical resection after neoadjuvant chemotherapy as the standard treatment for borderline resectable pancreatic cancer (BRPC) [7,8]. While the survival benefits of surgical resection after neoadjuvant treatment (NAT) are well established, not all patients undergoing NAT achieve successful conversion surgery in real-world clinical practice. To definitively establish the survival advantages of NAT, it is essential to investigate its impact on patients who experience failed surgery. Most previous studies have focused on patients who underwent surgery following NAT [9,10,11,12,13]. Thus, a study evaluating the impact of guideline changes on actual clinical outcomes is necessary to assess the survival benefits.

This study aimed to investigate the actual survival benefit after changing the first-line treatment for BRPC. We identified the value of NAT for survival outcomes in patients with BRPC.

## 2. Materials and Methods

### 2.1. Patient Database

After obtaining approval from the Institutional Review Board (IRB) (No. 2022-08-108, 17 August 2022 approved), we reviewed baseline computed tomography (CT), and magnetic resonance imaging (MRI) findings of all patients diagnosed with pancreatic ductal adenocarcinoma (PDAC) at the Samsung Medical Center during 2013–2015 and 2017–2019. Patients treated within the period of the guideline change in 2016 were excluded. Finally, 165 patients met retrospective BRPC criteria according to NCCN guidelines. BRPC was defined as: (1) solid tumor contact with the superior mesenteric artery and/or celiac artery (CA) of less than 180°; (2) solid tumor contact or infiltration into the common hepatic artery with an intact and uninvolved CA and/or proper hepatic artery; (3) solid tumor contact with the superior mesenteric vein of 180° or more, contact of less than 180° with contour irregularity of the vein or thrombosis of the vein, but allowing for safe and complete resection and vein reconstruction [14].

### 2.2. Classification of Patients

**Figure 1:**
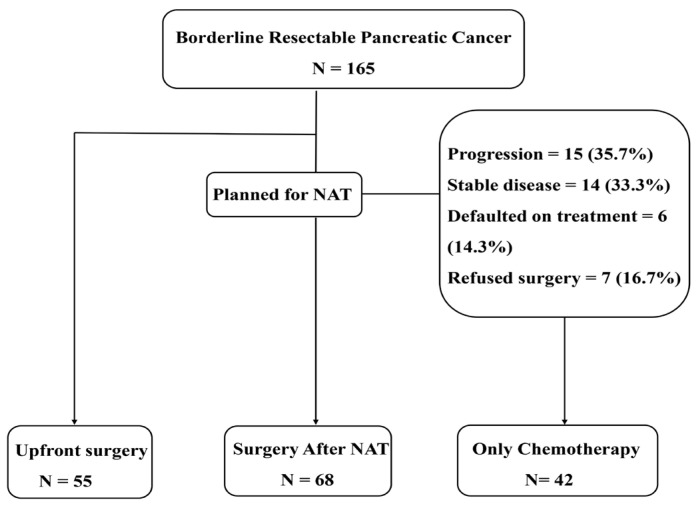
Flow chart of the study. NAT—neoadjuvant treatment.

We categorized patients into upfront surgery (US), surgery after neoadjuvant treatment (NAT), and chemotherapy only (CO) groups according to the treatment type. Patients from 2013 to 2015 and 2017 to 2019 were assigned to the pre- and post-guideline groups, respectively (Figure 1).

Their demographic information, pretreatment laboratory and imaging findings, pathology reports, and survival outcomes were retrospectively reviewed. Postoperative complications were documented according to the standard Clavian–Dindo classification [15]. Pancreas-specific complications such as delayed gastric emptying (DGE), post-pancreatectomy hemorrhage (PPH), and postoperative pancreatic fistula (POPF) were recorded with a grade of B/C according to the International Study Group of Pancreatic Surgery (ISGPS) definitions [16,17,18]. Detailed information on the NAT is shown in a supplementary data table (Table S1). Follow-up data were retrieved until December 2022.

### 2.3. Statistical Analysis

Overall survival (OS) was calculated from the date of the first treatment to the date of death or the last follow-up date. Disease-free survival (DFS) was calculated from the date of surgery to the date of radiologically confirmed relapse. In the CO group, progression-free survival (PFS) was defined as the length of time from the start of chemotherapy to the date of change in regimen, which was used for comparison instead of DFS. The clinical characteristics of patients according to treatment period and method were compared using the chi-square or Fisher’s exact test, independent *t*-test, and analysis of variance (ANOVA). Survival estimates were plotted using the Kaplan–Meier method and compared using the log-rank test. The Cox proportional hazard model was used to identify prognostic factors associated with survival outcomes. Variables with *p*-values less than 0.05 were regarded as statistically significant. All statistical analyses were performed using SPSS Statistics for Windows, version 27 (IBM Corp. Chicago, IL, USA).

## 3. Results

### 3.1. Clinical Characteristics and Survival Outcomes

Among the 165 patients, we identified 55, 68, and 42 cases of US, NAT, and CO, respectively. Clinical characteristics, including tumor size, location, and extent of vessel involvement, which were identified in the pretreatment images, did not differ among the three groups (Table 1). The median follow-up duration was 44 months for NAT, 66 months for US, and 59 months for CO groups.

**Figure 2:**
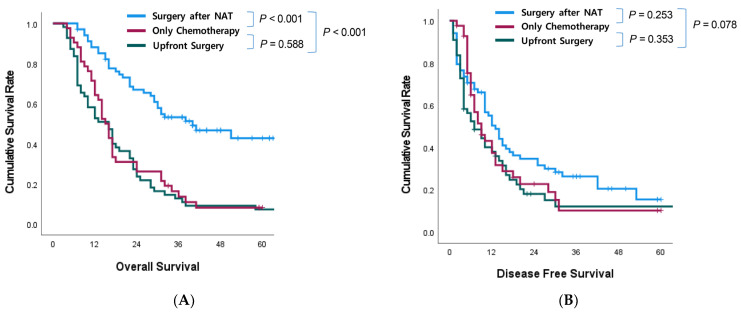
Survival analysis according to treatment type. (**A**) Overall survival depending on treatment type. (**B**) Disease-free survival depending on treatment type. NAT—neoadjuvant treatment.

The median OS of the NAT group was significantly longer than that of the US and CO groups (40 vs. 16 vs. 15 months, *p* < 0.001) (Figure 2A). However, there was no significant difference in median DFS among NAT, US, and CO groups, which had 13, 7, and 9 months, respectively (Figure 2B).

Based on their treatment periods, 63 patients were in the pre-guideline group and 102 were in the post-guideline group. A detailed comparison of the baseline characteristics of the two groups is presented in Table 2. There were no significant differences in most factors, including age, sex, and baseline tumor characteristics, as confirmed by preoperative images. Regarding the treatment method, the post-guideline group had more patients who received NAT than the pre-guideline group (100% vs. 12.7%, *p* < 0.001).

**Figure 3:**
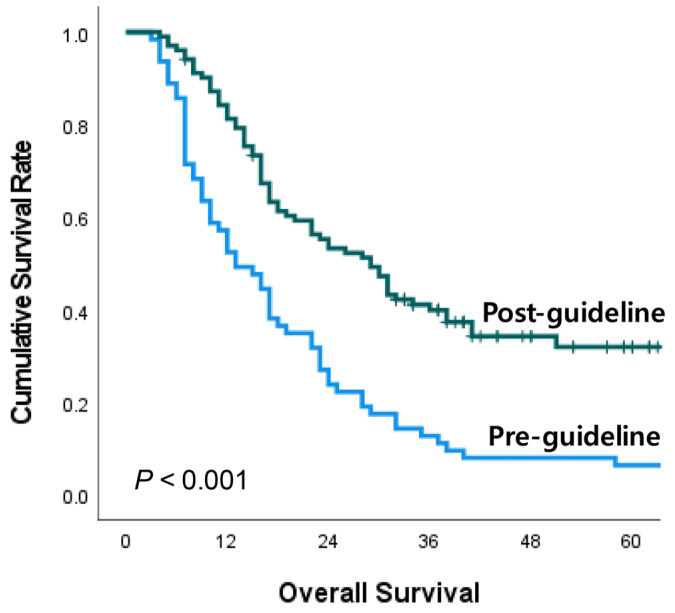
Survival analysis according to treatment period.

Figure 3 shows the OS curves for both groups. The median OS was significantly improved in the post-guideline group compared to the pre-guideline group (29 vs. 13 months, *p* < 0.001).

### 3.2. Clinicopathological Outcomes of Resected Patients

A comparison of the clinicopathological outcomes between the NAT and US groups is shown in Table 3. There was no significant difference between the two groups in terms of perioperative outcomes. The tumor size was significantly smaller in the NAT group than in the US group (23 ± 14 mm vs, 35 ± 11, *p* < 0.001). Patients in the NAT group had a lower incidence of vascular resection than those in the US group (41.2% vs. 63.6%, *p* < 0.013). In addition, the node-negative and R0 resection rate were higher in the NAT group (69.1% vs. 20.0%, *p* < 0.001; 79.4% vs.52.7%, *p* < 0.001). The incidences of perineural invasion (PNI) and lymphovascular invasion (LVI) were significantly lower in the NAT group than in the US group (69.1% vs. 100%, *p* < 0.001; 35.3% vs. 69.1%, *p* < 0.001).

### 3.3. Prognostic Factors Analysis

Risk factor analysis was performed to investigate the factors associated with OS in patients who underwent resection for BRPC (Table 4). In multivariate analysis, tumor size, differentiation, NAT, and PNI were statistically significant prognostic factors (hazard ratio (HR) = 1.039; 95% confidence interval (CI): 1.019–1.059; *p* < 0.001; HR = 0.258; 95% CI: 0.154–0.434, *p* < 0.001; HR = 0.454; 95% CI: 0.248–0.830, *p* = 0.01; and HR = 2.303; 95% CI: 1.014–5.231, *p* = 0.046, respectively).

## 4. Discussion

The shift towards NAT as the first-line treatment for BRPC has resulted in improved survival outcomes for many patients. Several studies have indicated that patients who undergo surgery after NAT have higher OS rates than those who undergo surgery first, thus supporting the benefits of NAT [9,10,11,12,13]. Nonetheless, research on the survival outcomes of patients who are unable to undergo surgical resection after NAT is limited. Not all patients achieve a successful surgical conversion after NAT in the real world. Therefore, it is important to understand the real-world impact of NAT in patients with BRPC, including those who fail surgery after undergoing chemotherapy.

The present study demonstrated the superior median OS of surgery after NAT compared to upfront surgery. Notably, the group that did not achieve conversion surgery after NAT had similar median OS and DFS rates as the group that underwent upfront surgery. Despite concerns about the potential missed opportunity for surgery after NAT, our findings suggest that NAT can offer a window of surgical opportunity, while maintaining a survival rate similar to that of immediate surgery. Regarding the treatment period, a significant improvement in the median OS rate of BRPC patients was observed, increasing from 13 to 29 months after the guideline change. The proportion of patients who received NAT as the first-line treatment surged notably from 12.7% to 100%. These results indicate that the increased utilization of NAT plays a major role in enhancing real-world survival outcomes. Our study, investigating all patients treated for BRPC at single institution, demonstrated an increased survival rate, even when including those who experienced failed surgical conversion after NAT. This suggests actual survival benefit in the real clinical world resulting from the guideline change.

In our study, the rate of conversion surgery was 61.8%, which is consistent with the results of previous meta-analyses that reported conversion rates of 65.3–67% [19,20]. The major reason for the failure to attempt surgical resection was the absence of changes in imaging findings. Post-NAT imaging using the Response Evaluation Criteria in Solid Tumors (RECIST) validated the presence of stable disease in 65.5% of patients (Table S1). However, previous studies have shown that imaging findings after NAT may not reliably predict tumor resectability, suggesting that the actual number of resectable cases may have been higher. The 2022 NCCN Guidelines suggest that exploration should be considered when there is a lack of clear progression on post-NAT imaging [21,22]. Therefore, the benefits of NAT are more significant than the potential risks associated with delayed surgery.

Several studies have reported that NAT has the potential to downstage tumors [15,23,24,25]. In our study, we compared the outcomes between the NAT and US groups. There was no difference in the tumor size or the extent of vessel involvement, as confirmed by imaging at the time of diagnosis. However, a comparison of pathological outcomes showed that the NAT group had more patients with no nodes, smaller tumor sizes, and fewer cases of vascular resection. Moreover, we observed that five patients in the NAT group achieved complete tumor remission. This suggests that NAT can effectively shrink tumors, allowing for a higher rate of complete tumor resection. The R0 resection rate was higher in the NAT group, which could be attributed to the benefits of NAT.

Another important finding of this study is that the NAT group had significantly lower PNI and LVI rates than the US group. Pancreatic cancer cells usually grow along nerves in contact with lymph nodes, suggesting that the PNI may be a route for lymph node metastasis [26]. In particular, PNI has been reported to have adverse effects on both OS and DFS in patients who have undergone R0 resection. Some studies identified PNI as an independent predictor of tumor recurrence, particularly in the R0/N0 subgroup [27,28]. LVI is also a crucial pathological feature that predisposes patients to regional lymph node metastasis and hematogenous spread [29,30]. Therefore, while experienced surgeons may argue that high-skilled surgery can achieve R0 resection in BRPC, micrometastases via PNI and LVI may persist. As a result, even successful R0 resection can still negatively affect the prognosis of patients with BRPC.

We conducted multivariate analysis to identify the factors that influence the survival rate of patients with BRPC after surgery. The analysis revealed that tumor size, differentiation, and the presence of PNI and NAT were independent factors affecting survival. Histologic differentiation is recognized as a significant prognostic factor. Well differentiated PDAC is associated with long-term survival of over 5 years, whereas poor histological differentiation is widely known as worse prognostic factor, often coexisting with other unfavorable indicators such as PNI, LVI, and node positivity [31,32]. Although our study did not demonstrate a significant impact of NAT on histologic differentiation, the meta-analysis on the effects of NAT reported that it tends to decrease the proportion of poorly differentiated PDAC [24]. Therefore, considering the significance of histologic differentiation and the demonstrated tumor down-staging effect of NAT, along with its impact in reducing rates of PNI, it can be concluded that NAT is a pivotal factor for improving the prognosis of patients with BRPC.

The survival benefit of NAT showed a clear advantage over upfront surgery; however, in terms of DFS, NAT had little effect on survival. Although the DFS was higher in the NAT group than in the US group, the difference was not statistically significant. However, our study had a relatively small sample size and a short follow-up duration, which may have contributed to this result. Nevertheless, previous studies have reported better DFS outcomes in the NAT group than the upfront surgery group [33,34]. Therefore, although our study did not observe a statistically significant difference in DFS between the NAT and US groups, the benefits of NAT in terms of DFS cannot be ruled out and should be investigated in future studies with larger sample sizes and actual 5-year survival outcomes.

This study has a few limitations worthy of discussion. First, in this study, we utilized the anatomic criteria for defining BRPC based on NCCN guidelines, and we did not consider the elevated cancer antigen 19-9 as a biologic condition. There could have been selection bias during the review of CT and MRI scans for BRPC. Second, comparing survival rates across different periods cannot solely be attributed to the increase in NAT, as external factors such as improved surgical expertise and advancements in imaging techniques might also be involved. However, our study had the advantage of accessing detailed information from a single institution, which reduced selection bias compared with previous meta-analyses or national cancer database studies. Therefore, we assessed real-world survival outcomes after changing guidelines for BRPC. Furthermore, our study presents the latest clinical outcomes, since we analyzed patients treated between 2017 and 2019.

In conclusion, this study demonstrated that NAT is an important treatment option for BRPC and has contributed to increased survival in the real world. NAT provides an opportunity for surgery by down-staging tumors and improving survival outcomes, making it an important treatment option for patients with BRPC.

## Figures and Tables

**Table 1:** Comparison of baseline characteristics according to treatment type.

Variables	Upfront Surgery (*n* = 55)	Surgery after NAT (*n* = 68)	Only Chemotherapy (*n* = 42)	*p*
Age, year, mean ± SD	63 ± 10	61 ± 10	64 ± 8	0.254
Sex, *n* (%)				0.214
Male	27 (49.1)	40 (58.8)	28 (66.7)	
Female	28 (50.9)	28 (41.2)	14 (33.3)	
Tumor location, *n* (%)				0.157
Head	39 (70.9)	48 (70.6)	36 (85.7)	
Body/tail	20 (29.1)	20 (29.4)	6 (14.3)	
Tumor size, mm (%)				0.126
≤25	14 (25.5)	15 (22.1)	6 (14.3)	
>25–30	13 (23.6)	6 (8.8)	10 (23.8)	
>30–35	15 (27.3)	19 (27.9)	14 (33.3)	
>35	13 (23.6)	28 (41.2)	12 (28.6)	
Vessel involvement, *n* (%)				0.27
Venous	40 (72.7)	41 (60.3)	30 (71.4)	
Arterial	10 (18.2)	13 (19.1)	4 (9.5)	
Both	5 (9.1)	14 (20.6)	8 (19)	
Serum CA19-9, U/mL, *n* (%)				0.263
<500	42 (76.4)	50 (73.5)	26 (61.9)	
≥500	13 (23.6)	18 (26.5)	16 (38.1)	

NAT—neoadjuvant treatment; SD—standard deviation; CA19-9—cancer antigen 19-9.

**Table 2:** Comparison of baseline characteristics according to treatment period.

Variables	2013–2015 (*n* = 63)	2017–2019 (*n* = 102)	*p*
Age, year, mean ± SD	63 ± 10	62 ± 9	0.576
Sex, *n* (%)			0.087
Male	31 (49.2)	64 (62.7)	
Female	32 (50.8)	38 (37.3)	
Tumor location, *n* (%)			
Head	44 (69.8)	79 (77.5)	0.276
Body/tail	19 (30.2)	23 (22.5)	
Tumor size, mm (%)			0.189
≤25	15 (23.8)	20 (19.6)	
>25–30	15 (23.8)	14 (13.7)	
>30–35	18 (28.6)	30 (29.4)	
>35	15 (23.8)	38 (37.3)	
Vessel involvement, *n* (%)			0.079
Venous	43 (68.3)	68 (66.7)	
Arterial	14 (22.2)	13 (12.7)	
Both	6 (9.5)	21 (20.6)	
Serum CA19-9, U/mL, *n* (%)			0.161
<500	49 (77.8)	69 (67.6)	
≥500	14 (22.2)	33 (32.4)	
Treatment type			<0.001
Upfront surgery	55 (87.3)	0	
Neoadjuvant treatment	8 (12.7)	102 (100)	
Chemotherapy	0	100	
Chemo-radiotherapy	6	2	
Radiotherapy	2	0	

**Table 3:** Details on clinicopathological outcomes of resected patients.

Variables	Upfront Surgery (*n* = 55)	Surgery after NAT (*n* = 68)	*p*
Length of stay, day, median	10 (9–12)	10 (9–14)	0.914
Any Complications, *n* (%)	31 (56.4)	33 (48.5)	0.387
Higher than grade III complication, *n* (%)	11 (20)	11 (16.2)	0.582
POPF, *n* (%)	6 (10.9)	4 (5.9)	0.340
DGE, *n* (%)	3 (5.5)	5 (7.4)	0.730
PPH, *n* (%)	3 (5.5)	6 (8.8)	0.730
Tumor size, mm, mean ± SD	35 ± 11	23 ± 14	<0.001
Vascular resection, *n* (%)			0.013
No	20 (36.4)	40 (58.8)	
Yes	35 (63.6)	28 (41.2)	
T stage, *n* (%)			<0.001
0	0	5 (7.4)	
1	5 (5.5)	24 (35.3)	
2	36 (65.5)	33 (48.5)	
3	16 (29.1)	4 (5.9)	
4	0	2 (2.9)	
N stage, *n* (%)			<0.001
0	11 (20.0)	47 (69.1)	
1	26 (47.3)	14 (20.6)	
2	18 (32.7)	7 (10.3)	
Margin status, *n* (%)			<0.001
R0	29 (52.7)	54 (79.4)	
R1/R2	26 (47.3)	14 (20.6)	
Tumor differentiation, *n* (%)			0.071
Well differentiated	2 (3.6)	1 (1.6)	
Moderately differentiated	33 (60.0)	50 (79.4)	
Poorly differentiated	20 (36.4)	12 (19.0)	
Perineural invasion, *n* (%)			<0.001
No	0	21 (30.9)	
Yes	55 (100)	47 (69.1)	
Lymphovascular invasion, *n* (%)			<0.001
No	17 (30.9)	44 (64.7)	
Yes	38 (69.1)	24 (35.3)	

POPF, postoperative pancreatic fistula; DGE, delayed gastric emptying; PPH, post-pancreatectomy hemorrhage; NAT, neoadjuvant treatment; SD, standard deviation.

**Table 4:** Factors associated with overall survival in the resected patients with BRPC.

	Univariate Analysis		Multivariate Analysis	
Variables	Hazard Ratio (95% CI)	*p*	Hazard Ratio (95% CI)	*p*
Age, years				
<65	Ref.			
≥65	1.237 (0.802–1.907)	0.336		
Sex				
Male	Ref.			
Female	0.815 (0.531–1.249)	0.348		
Tumor location				
Head	Ref.			
Body/Tail	1.231 (0.776–1.952)	0.378		
CA19-9 normalization				
No	Ref.		Ref.	
Yes	0.543 (0.345–0.854)	0.008	1.467 (0.916–2.349)	0.111
Tumor size, mm	1.036 (1.021–1.051)	<0.001	1.039 (1.019–1.059)	<0.001
Vascular resection				
No	Ref.		Ref.	
Yes	1.678 (1.087–2.589)	0.019	1.125 (0.705–1.796)	0.621
Margin status				
Negative	Ref.		Ref.	
Positive	1.855 (1.202–2.862)	0.005	0.840 (0.515–1.371)	0.486
Lymph node status				
Negative	Ref.		Ref.	
Positive	2.360 (1.509–3.691)	<0.001	1.277 (0.703–2.320)	0.423
Tumor differentiation				
Poorly differentiated	Ref.	<0.001	Ref.	<0.001
Moderate differentiated	0.397 (0.094–1.671)	0.208	0.304 (0.070–1.310)	0.110
Well differentiated	0.354 (0.223–0.561)	<0.001	0.258 (0.154–0.434)	<0.001
Neoadjuvant treatment				
No	Ref.		Ref.	
Yes	0.306 (0.197–0.474)	<0.001	0.454 (0.248–0.830)	0.01
Perineural invasion				
No	Ref.		Ref.	
Yes	1.872 (0.993–3.529)	0.052	2.303 (1.014–5.231)	0.046
Lymphovascular invasion				
No	Ref.		Ref.	
Yes	1.831 (1.191–2.814)	0.006	1.153 (0.651–2.044)	0.625

BRPC—borderline resectable pancreatic cancer; CA19-9—cancer antigen 19-9; CI—confidence interval.

## Data Availability

All data generated in this study are unavailable due to the hospital’s privacy policy.

## Supplementary Materials

The following supporting information can be downloaded at: https://www.mdpi.com/article/10.3390/biomedicines11082302/s1, Table S1. Detailed information on neoadjuvant treatment.
