# Supplementary material for: Survival Benefit after Shifting from Upfront Surgery to Neoadjuvant Treatment in Borderline Resectable Pancreatic Cancer
Source: Biomedicines. 2023 Aug 18;11(8):2302. doi: 10.3390/biomedicines11082302 (PMC10452854; doi:10.3390/biomedicines11082302)
Supplement: Supplementary file 1 [file biomedicines-11-02302-s001.zip › biomedicines-2532009-supplementary.pdf]

Table S1 Detailed information on neoadjuvant treatment.

Variables	N (%)
Types of regimens	
FOLFIRINOX	91 (89.2)
Gemcitabin-nab-paclitaxel	9 (8.8)
CCRT	2 (2.0)
Cycle, median (IQR)	6 (4-10)
RECIST response	
Complete Response	1 (0.9)
Partial Response	30 (27.3)
Stable Disease	72 (65.5)
Progressive Disease	7 (6.4)
CA19-9 response†	
Yes	52 (47.3)
No	33 (30.0)
Initially not elevated	25 (22.7)
Conversion Surgery	68 (61.8)
Curative-intent Surgery	62 (56.3)

†Response was defined if the value measured after 4 cycles of chemotherapy was decreased by more than 50% from the initial.
